# Chest-Related Imaging Investigations During Multiple Waves of COVID-19 Infection in Hong Kong

**DOI:** 10.3389/fmed.2021.704515

**Published:** 2021-11-17

**Authors:** Kei Shing Ng, Varut Vardhanabhuti

**Affiliations:** Department of Diagnostic Radiology, Li Ka Shing Faculty of Medicine, The University of Hong Kong, Hong Kong, Hong Kong SAR, China

**Keywords:** COVID-19, X-ray, CT, chest ultrasound, interventional radiology

## Abstract

**Background:** The COVID-19 pandemic has caused significant disruption to healthcare worldwide. In this study, we aim to quantify its impact of chest related radiological procedures over the different waves of local infection in Hong Kong across the territory's public hospitals.

**Methods:** This was an observational study enrolling patients between January 2017 and December 2020. Consecutive population-based chest radiographs, CT, US, and interventional radiology (IR) procedures were obtained public hospitals across Hong Kong.

**Results:** A significant reduction of 10.0% (*p* < 0.001) in the total number of chest radiographs was observed. Non-significant reduction of 2.5% (*p* = 0.0989), 39.1% (*p* = 0.2135), and 1.9% (*p* = 0.8446) was observed for Chest CT, Chest US, and Chest IR procedures, respectively, in 2020 compared to the projected values.

**Conclusion:** Although, it was anticipated that there would be a significant impact to health services caused by the pandemic, for chest-related investigations in Hong Kong, the impact was not as severe. Quantitative analysis could help with future planning and public health decision making.

## Introduction

Significant impact of coronavirus disease 2019 (COVID-19) was observed in the year 2020. In particular, medical services have been reduced or have been redirected toward caring for patients with coronavirus disease 2019 (COVID-19) (1). The first local COVID-19 case in Hong Kong was reported on January 23, 2020. On October 18, 2021, 12,294 confirmed cases (2,952 imported cases and 9,342 local cases) and 213 deaths were reported in the territory. In terms of medical imaging, varying impacts on chest ultrasound (2) and interventional radiology (3–6) were reported globally, but few studies have examined the impact at a population level over a prolonged period. The effects of chest X-ray and chest CT have also not been quantitatively reported at a population scale. In this study, we systematically examined territory-wide chest-related imaging investigational data in the Hong Kong public hospitals database and compare the impact in 2020 with the prior 3 years.

## Methods

The Hong Kong Hospital Authority Clinical Data Analysis and Reporting System (CDARS) was used to conduct a retrospective search of electronic records of patients using a national database. The study was approved by the institutional review board, and, due to the retrospective nature of the study, the patient consent was not required. The study retrieved data between January and December of the years 2017 and 2020, covering chest imaging investigations, specifically chest radiographs, chest computed tomography (CT), chest ultrasound (US), and chest interventional radiology (IR) procedures. The time series model was conducted on the data from the prior years of 2017–2019 to estimate the predicted number of procedures in 2020 (Time Series Modeller, SPSS, IBM Corp., Armonk, N.Y., USA). Differences in counts between the predicted and actual values were compared using a generalised linear model with the Poisson distribution. Statistical significance was set as *p* < 0.05. The analysis was performed using R Studio version 1.3 (rstudio.com, Boston, Massachusetts, United States).

## Results

**Figure 1:**
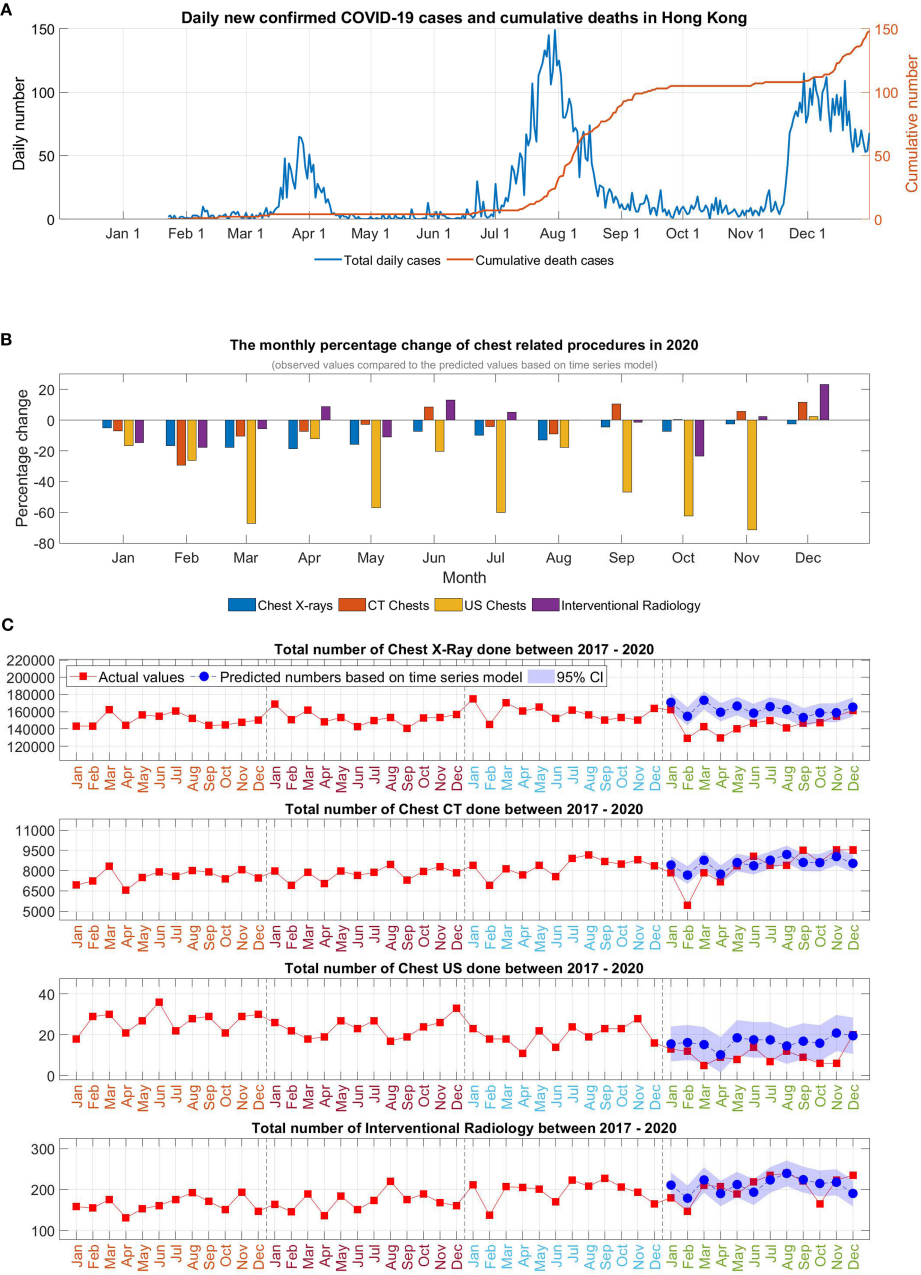
**(A)** Daily newly confirmed COVID-19 cases and cumulative deaths in Hong Kong, **(B)** The monthly percentage change of chest-related procedures in 2020, **(C)** Time series analysis of the chest-related procedures in 2020.

In 2020, there were a total number of 1,751,881 chest radiographs, 99,692 chest CT, 121 chest US, and 2,472 chest IR procedures performed from 35, 19, 11, and 18 hospitals, respectively, across public hospitals in Hong Kong. A significant reduction of 10.% (*p* < 0.001) in the total number of chest radiographs was observed in 2020 compared to the predicted value based on the time series model. Non-significant reduction of 2.5% (*p* = 0.0989), 39.1% (*p* = 0.2135), and 1.9% (*p* = 0.8446) was observed for Chest CT, Chest US, and Chest IR procedures in 2020 compared to the predicted values based on the time series model (see Table 1, Figure 1).

**Table 1:** The actual, predicted number using the time series method and percentage change of chest-related procedures in 2020.

**2020**	**Chest X-rays**			**CT Chests**			**US Chests**			**Interventional Radiology**		
	**Actual**	Predicted^*^	**% change**	**Actual**	Predicted^*^	**% change**	**Actual**	Predicted^*^	**% change**	**Actual**	Predicted^*^	**% change**
Jan	162,185	170,811	−5.05	7,828	8,413	−6.96	13	16	−16.51	180	211	−14.65
Feb	129,066	154,692	−16.57	5,429	7,668	−29.20	12	16	−26.11	147	179	−17.83
Mar	142,525	173,219	−17.72	7,848	8,761	−10.42	5	15	−67.19	211	224	−5.61
Apr	129,669	159,359	−18.63	7,182	7,739	−7.20	9	10	−12.11	207	190	8.82
May	140,276	166,580	−15.79	8,354	8,594	−2.80	8	19	−56.92	189	212	−10.94
Jun	146,924	158,344	−7.21	9,065	8,361	8.43	14	18	−20.32	219	194	13.15
Jul	149,828	165,895	−9.69	8,403	8,770	−4.18	7	18	−60.16	235	224	5.12
Aug	141,393	162,393	−12.93	8,372	9,195	−8.96	12	15	−17.64	240	240	0.19
Sep	146,512	153,307	−4.43	9,513	8,601	10.60	9	17	−46.78	221	224	−1.44
Oct	147,334	158,694	−7.16	8,624	8,587	0.43	6	16	−62.29	165	215	−23.22
Nov	154,940	158,854	−2.46	9,540	9,035	5.59	6	21	−71.31	223	218	2.35
Dec	161,229	165,331	−2.48	9,534	8,535	11.70	20	20	2.20	235	191	23.33
Total	1,751,881	1,947,479	−10.04	99,692	102,261	−2.51	121	199	−39.16	2,472	2,520	−1.90
Stationary R–squared		0.727			0.814			0.836			0.843	
R–squared		0.650			0.742			0.446			0.684	
RMSE		5148.005			318.861			4.215			15.162	
MAPE		2.562			3.056			15.704			6.823	
MAE		3930.920			239.558			3.384			11.893	
MaxAPE		10.175			8.505			49.340			17.637	
MaxAE		14593.800			616.880			8.420			34.263	
Normalised BIC		17.391			11.828			3.176			5.736	

*RMSE, Root mean square error; MAE, Mean absolute error; MaxAPE, Maximum absolute percentage error; MaxAE, Maximum absolute error; Normalised BIC, Normalised Bayesian information criterion*.

* *The predicted values were rounded to nearest integer, and the percentage change was based on the full digits of the predicted value*.

Monthly analysis revealed that there was an overall trend of a marked initial reduction in the early months at the start of pandemic. Cases were returned to almost a pre-pandemic level during the easing of social restrictions but reduced again in July 2020, coinciding with the reemergence of infection.

For chest radiograph, reduction was observed in all the months of 2020 compared to predicted numbers (with negative % in all the months). For chest CT, although marked reduction was observed initially, there was an increase in number of investigations in the months of June, and September to December, and, by the year end, there was only minimal overall reduction of 2.5% for 2020. For chest US, the monthly changes showed a double-digit percentage reduction in January to November (−12.1 to −71.3%) in 2020 with only a mild catchup (2.2%) in December. For chest IR, monthly analysis showed a reduction in the period of January to March in 2020, and subsequently increased in the following months with another reduction in October, with increasing numbers again toward year end. As a result, the overall yearly reduction was mild (−1.9%, *p* = 0.8446).

**Figure 2:**
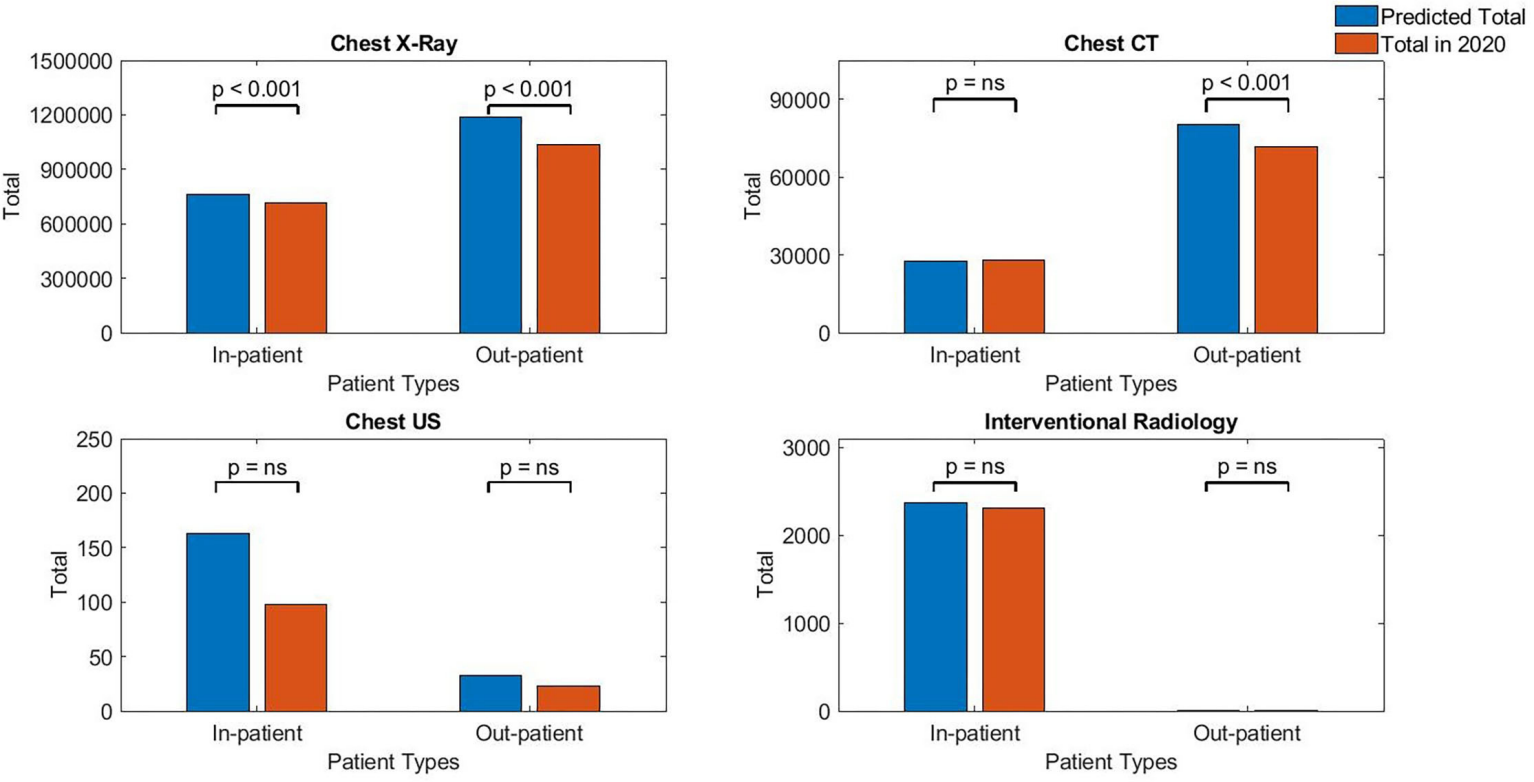
Chest-related investigations for different patient types in 2020.

When analysis was done based on patient types, for chest radiographs, the impact was more for outpatient investigations (−12.45%, *p* < 0.001) compared with inpatient (−6.40%, *p* < 0.001). For Chest CT, outpatient investigations were more affected with overall reduction observed (−11.05%, *p* < 0.001) compared to mild increase for inpatient investigations (+2.7%, *p* = 0.3716). For Chest US, the inpatient contributed a marked reduction (−39.8%, *p* = 0.2516) compared to a modest reduction for outpatient (−28.7%, *p* = 0.7210). Finally, for chest IR, the reduction by type of a patient was mild for inpatient (−2.7%, *p* = 0.7882) and large for outpatient (−50.7%, *p* = 0.7720). However, the total chest IR done for outpatient was <10 procedures, which may be regarded negligibly small to draw firm conclusion (see Figure 2, Table 2).

**Table 2:** The numbers of inpatient vs. outpatient vs. total number using the time series method for all four imaging investigations.

	**Chest X–rays**			**CT Chests**			**US Chests**			**Interventional radiology**		
	**Inpatient**	**Outpatient**	**Total**	**Inpatient**	**Outpatient**	**Total**	**Inpatient**	**Outpatient**	**Total**	**Inpatient**	**Outpatient**	**Total**
2020 Actual	713,161	1,038,720	1,751,881	28,177	71,515	99,692	98	23	121	2,312	3	2,472
2020 Predicted^*^	761,895	1,186,389	1,947,479	27,447	80,400	102,261	163	32	199	2,376	6	2,520
% change	−6.40	−12.45	−10.04	2.66	−11.05	−2.51	−39.82	−28.66	−39.20	−2.68	−50.74	−1.90

* *The predicted values were rounded to nearest integer, and the percentage change was based on the full digits of the predicted value*.

## Discussion

It was perceived that many radiology departments may observe up to a 50–70% reduction in imaging volume that may last for several months during the COVID-19 pandemic (7). In our study, during the initial waves, a more pronounced reduction was observed, which was most likely attributed to a tighter initial lockdown and cancellation of procedures, likely due to a more conservative stance taken due to a relative lack of knowledge about the new disease. The conservative stance may have stemmed from a previous encounter of Hong Kong with the SARS outbreak in 2003 (8), when the mortality rate in Hong Kong was 17.04%, which was about three times that of China (4.87%). Original findings of a high infection incidence and a mortality rate (2.97%) among healthcare staff in hospitals (9), as well as a lack of protective gear for hospitals and clinics (10), may have contributed to the extreme vigilance of the government and refusal of patients to attend hospital appointments (11). Anecdotally, this was often found at the local level, where some patients would phone to postpone or cancel appointments.

Despite of the confirmed case numbers being much higher for the subsequent waves of infection in December 2020, the opposite trend was observed with only a minor decline in Chest X-ray and a small increase in CT Chest, US Chest, and IR. This was more likely due to improved preparedness and increased precautions implemented in hospitals as well as more experience in coping with the COVID-19 pandemic in the months following the outbreak. During these times, precautions, such as wearing masks, mandatory measurement of body temperature, protective covering for staffs, and frequent hand washing, were in place. The adoption of COVID-19 contact tracing apps for patients and anti-pandemic legislation Cap. 599 on home quarantine and social distancing, and compulsory testing for certain persons introduced by the Hong Kong SAR government were also routine procedures with a high adoption rate in Hong Kong.

There were likely multiple reasons for the reduction in radiological procedures observed. The reason for less impact on chest CT and chest IR procedures may be postulated to be due to the fact that more severely ill patients still required these crucial investigational procedures. In addition, we postulate that there were increasing needs of cross-sectional imaging and interventional procedure requirement for chest-related diseases during the pandemic. There may also have been preferential imaging practises, which caused a reduction of ultrasound procedure, for example, to try to minimise direct patient contact. This may be the reason why there was a reduction in chest US inpatient numbers (although not significantly reduced). It must be noted that the numbers were few, in general, to draw any firm conclusion. It was not possible to clearly identify the reasons (e.g., if this was due to change of practise or behaviour of physicians) due to retrospective nature of the study across multiple sites. Looking ahead, as we move toward living with COVID-19 as endemic infections, continued practises of enhanced precautions may become permanent. Health providers must, therefore, try to be more proactive and anticipate potential impact in the event of an outbreak, and minimise the impact of reduction in radiological investigations as far as possible. There are a few ways that one could do this. First, for urgent investigations, provisions must be put in place to be able to provide these services and minimise the cancellations as far as possible. Second, if cancellations were to occur for non-urgent cases, the healthcare providers should adopt a system that allows for a more rapid rearrangement and, for example, may increase the quota of non-emergency outpatient investigations to offset any reduction of services during the localised outbreaks. Radiologists should work closely with their referral doctors to review and reschedule such examinations.

In conclusion, despite relatively low case numbers in Hong Kong compared to other countries, a significant reduction in chest radiographs investigation was seen, but not for chest CT, chest US, or chest IR procedures when analysed at a population level based on yearly analysis.

## Data Availability Statement

The raw data supporting the conclusions of this article will be made available by the authors, without undue reservation.

## Ethics Statement

The studies involving human participants were reviewed and approved by Cluster Research Ethics Committee/Institutional Review Board (REC/IRB). Written informed consent for participation was not required for this study in accordance with the national legislation and the institutional requirements.

## Author Contributions

KN and VV were involved in study conception, statistical analysis, drafting of the initial version of the manuscript, data collection, and statistical analysis. VV supervised the work and offered significant intellectual contribution. All the authors offered significant intellectual contribution for the last version of the manuscript and approved the final form.

## Conflict of Interest

The authors declare that the research was conducted in the absence of any commercial or financial relationships that could be construed as a potential conflict of interest.

## Publisher's Note

All claims expressed in this article are solely those of the authors and do not necessarily represent those of their affiliated organizations, or those of the publisher, the editors and the reviewers. Any product that may be evaluated in this article, or claim that may be made by its manufacturer, is not guaranteed or endorsed by the publisher.
